# Cold, Hot, and Lethal—The Tumour Microenvironment and the Immunology of Head and Neck Squamous Cell Carcinoma

**DOI:** 10.3390/ijms26188844

**Published:** 2025-09-11

**Authors:** Svatava Vyhnánková, Lukáš Lacina, Martin Chovanec, Jan Plzák, Karel Smetana, Jiří Netušil, Michal Kolář, Radek Šindelka

**Affiliations:** 1 Department of Otorhinolaryngology 3FM CU and UHKV, Charles University, 10034 Prague, Czech Republic; svatava.vyhnankova@fnkv.cz (S.V.); martin.chovanec@fnkv.cz (M.C.); 2 Institute of Anatomy of the 1st Faculty of Medicine, Charles University, 12800 Prague, Czech Republic; karel.smetana@lf1.cuni.cz; 3 General University Hospital, 12800 Prague, Czech Republic; 4 Department of Otorhinolaryngology, Head and Neck Surgery First Faculty of Medicine Charles University and University Hospital Motol, 15000 Prague, Czech Republic; jan.plzak@motol.cz; 5 Institute of Molecular Genetics of the Czech Academy of Sciences, 14200 Prague, Czech Republic; jiri.netusil@img.cas.cz (J.N.); michal.kolar@img.cas.cz (M.K.); 6 Faculty of Science, Charles University, 12800 Prague, Czech Republic; 7 Laboratory of Gene Expression, Institute of Biotechnology of the Czech Academy of Sciences, 25250 Vestec, Czech Republic; radek.sindelka@ibt.cas.cz

**Keywords:** head and neck squamous cell carcinoma, cancer, stroma, cancer-associated fibroblast, tumour microenvironment, immunity, therapy, IL-6, CAF, extracellular matrix

## Abstract

Head and neck squamous cell carcinomas (HNSCCs) represent a diverse group of malignancies, both clinically and biologically, with human papillomavirus (HPV) infection playing a significant role. HPV-positive tumours generally tend to have a better prognosis and are driven by oncoproteins E6 and E7. In contrast, HPV-negative tumours typically have a worse prognosis and are often linked to mutations in tumour suppressor genes. HNSCCs exist within a complex environment known as the tumour microenvironment (TME). The TME includes tumour cells, cancer stem cells (CSCs), cancer-associated fibroblasts (CAFs), immune cells, extracellular matrix (ECM), blood vessels, and various signalling molecules. These components support tumour progression, invasion, metastasis, and resistance to treatment. Intercellular signalling within the TME—mediated by cytokines such as IL-6, TGF-b, and galectins—further promotes tumour growth and systemic effects like cachexia. Notably, the TME shares features with granulation tissue during wound healing, supporting the concept of cancer as a chronic, non-resolving wound. Effective therapy must target not only tumour cells but also the dynamic TME.

## 1. Introduction

Head and neck squamous cell carcinomas (HNSCC) are the sixth most common cancers worldwide, with 50–70% of cases occurring in men aged 60–70 years. They originate from the squamous cell epithelium in the oral and nasal cavities, pharynx, larynx, and skin [1]. HNSCC risk factors include tobacco, alcohol, and human papillomavirus infection (HPV). Based on HPV status, HNSCC can be classified as either HPV-positive or HPV-negative [2]. Information about HPV status, alongside TNM staging (tumour, node, metastasis), serves as the primary prognostic factor for HNSCC. Generally, HPV-positive tumours tend to have a better prognosis. Notably, up to 85% of HPV-positive cases contain HPV-16 or HPV-18.

The pathogenesis of HNSCC primarily involves inhibition of tumour suppressor genes or their proteins (tumour suppressor proteins), particularly p53 and pRb. Their inactivation allows cells to bypass cell-cycle checkpoints, which also usually leads to the accumulation of DNA errors or mutations, and ultimately, these cells can undergo malignant transformation. In HPV-positive tumours, p53 (protein p53) and pRb (Retinoblastoma protein) are inhibited by viral oncoproteins E6 and E7. HPV-positive tumours are also linked with an upregulated production of p16 (protein p16), an immunohistochemical marker routinely used to detect HPV infection. Conversely, in HPV-negative tumours, p53 is inactivated by gene mutation, while pRb is inhibited by cyclin D1 or cyclin D2 and cyclin-dependent kinases (CDK-)4-6 complexes. HPV-negative tumours often exhibit deletion of the CDKN2A gene, which paradoxically correlates with reduced p16 expression and worse prognosis in comparison to HPV-positive tumours [3].

Tumours are highly heterogeneous complexes with structures that vary depending on the specific tumour type. Generally, genetically abnormal malignant cells are surrounded by complex environmental components that constitute what is known as the tumour microenvironment (TME). The TME is crucial for cancer cells; for example, it promotes cancer cell proliferation and migration, which may also contribute to metastatic progression. Moreover, this microenvironment consists of cancer cells, cancer-associated fibroblasts (CAFs), immune cells, endothelial cells, extracellular matrix (ECM), cytokines, chemokines, growth factors, and other elements [4]. These components not only facilitate intercellular tumour communication but also exert systemic effects through molecules such as TNF-α (Tumour Necrosis Factor), TGF-β (Transforming Growth Factor), IL-6, IL-10, and others [5]. Recent studies highlight the increasing expression of proteins that can recognise sugars, such as lectins and their glyco-ligands, in malignancies [6]. These glycoproteins (lectins) can interpret information hidden within structural carbohydrate motifs. Galectins (Gal), which bind beta-galactosides and poly-N-acetyllactosamines, have complex roles: they can regulate pre-mRNA splicing, proliferation, and apoptosis, and facilitate interactions between cells and the ECM. Additionally, galectins are involved in innate immunity. The deregulation of lectin and glyco-ligand (carbohydrate) expression significantly impacts the malignant transformation of the normal squamous epithelium and its differentiation, metastatic potential, and responsiveness to treatment [7]. The so-called glyco-code is a fundamental aspect of tumour biology, encoding biological information through monosaccharide sequences that form oligo- and polysaccharides [8].

With respect to the immunological activity estimated by the spatial distribution of T cells observed in solid tumours, three distinct immune phenotypes are currently recognised [9,10]. The first are inflamed/hot tumours, characterised by abundant intratumoural T cell infiltration. Next are desert/cold tumours, defined by minimal T cell presence. Lastly, there are immune-excluded tumours, in which even rich CD8^+^ T cells are evident; however, there is an obvious sequestration of these antitumoural lymphocytes within the tumour stroma only.

Over the past decade, pioneering immunotherapeutics, such as anti-CTLA4 (cytotoxic T lymphocyte-associated protein 4) and anti-PD-1 (programmed cell death protein 1) antibodies, have dramatically transformed the landscape of oncology. Immune checkpoint inhibitors (ICIs) have revolutionised cancer treatment. However, durable responses are achieved only in a subtype of treated patients. Primary or acquired resistance to these treatments is frequently seen. Sadly, even if immune cells seem to be abundant in biopsy, tumours can be or become resistant to ICIs due to upregulation of alternative checkpoints (TIM3 (T cell immunoglobulin and mucin-domain containing-3), LAG3 (lymphocyte-activation gene 3), and TIGIT (T cell immunoreceptor with Ig and ITIM domains)) and/or by using non-PD-1/CTLA4-mediated inhibitory pathways [11,12].

In this review, we aim to summarise the immunological factors of TME in HNSCC and elucidate the mechanisms of resistance of these tumours to oncological treatments. Emerging evidence highlights the role of CAFs in orchestrating the immunomodulatory aspects of TMEs, making CAFs the principal contributors to immune evasion and cancer therapy. We believe that research on the TME, and particularly on CAF heterogeneity, may ignite innovative strategies and can even boost the success of existing immunotherapies.

### 1.1. Tumour Stem Cells

Tumour cell heterogeneity is notable even within a single tumour in one patient, mainly due to diverse gene mutations, particularly so-called driver mutations, which are the primary cause of carcinogenesis initiation. Among these cells, there is a subpopulation that exhibits stem-like properties, known as cancer stem cells (CSCs) [13]. CSCs are poorly differentiated cells that are vital for tissue maintenance and regeneration. They have a distinctive localisation within tumour stroma, surrounded by a specific environment (niche) necessary for their normal function [14]. CSCs are capable of constant cell division, which supports tissue upkeep. Their division is very slow and asymmetric, producing one daughter cell that retains stemness and another that further differentiates. CSCs can actively exclude xenobiotics, including chemotherapeutic agents, from their cytoplasm, often rendering them resistant to conventional cancer therapies [15]. CSCs have been identified even in HNSCC [16,17], and they are implicated in minimal residual disease, tumour recurrence, metastasis, and drug resistance [18]. Somatic mutations accumulating with ageing, combined with impaired DNA repair, drive extensive mutagenesis. As a result, metastatic cells often possess a genetic profile distinct from their primary tumour counterparts. Tumour progression follows a Darwinian evolutionary trajectory, shaped by mutational landscape and microenvironment interactions [19,20]. Therefore, effective oncological therapy should target CSCs directly—either by eliminating their stemness or by modulating the TME to suppress tumour-promoting signals.

So what makes CSCs hot, cold, or lethal? Due to their slow population kinetics, CSCs are naturally resistant to conventional chemotherapy and radiotherapy. Considering novel immunotherapeutic options, immune evasion is a major obstacle for successful cancer treatment in many patients. Why do even these trendy therapeutic modalities fail?

It is well documented that immune privilege is not a general quality of all stem cells [21]. However, it has been confirmed that Lgr5^+^ stem cells of the bulge hair follicle in skin were entirely resistant to T cell attack, even by fully activated T cells [22]. The immune-privileged status of CSCs is induced and preserved by various mechanisms [23]. Common mechanisms of evasion include impaired antigen presentation caused by mutations or loss of heterozygosity of the major histocompatibility complex class I (MHC-I), which has been implicated in resistance to immune checkpoint inhibitor (ICI) therapy in some cancer types [24]. However, frequent downregulation of MHC-I expression seen in tumours is not always linked to such mutations. Alternatively, MHC-I molecules are in some tumours selectively targeted for lysosomal degradation by an autophagy-dependent mechanism. Notably, inhibition of autophagy, e.g., pharmacologically with the well recognised antimalarial drug chloroquine, restores surface levels of MHC-I and leads to improved antigen presentation [25]. This improves antitumour T cell responses, which can be enhanced therapeutically with anti-PD1 (programmed cell death protein 1 ligand) and anti-CTLA4 antibodies.

Using a model for cutaneous squamous cell carcinoma (SCC), tumours can be effectively challenged by adoptive cytotoxic T cell transfer (ACT)-based immunotherapy [26]. However, it was confirmed that transforming growth factor β (TGF-β)-responding CSCs are superior at resisting therapeutic T cell transfer. Mechanistically, CSCs selectively acquire CD80, a surface ligand previously identified on immune cells, and directly dampen cytotoxic T cell activity. Conversely, upon CTLA4- or TGF-β-blocking immunotherapies or CD80 ablation, CSCs become vulnerable, diminishing tumour relapse after ACT treatment. Therefore, deeper insights into the immunobiology of CSCs are essential in our pursuit to find new therapeutic opportunities that eradicate cancer cells, including CSCs.

### 1.2. Metastasis

**Figure 1:**
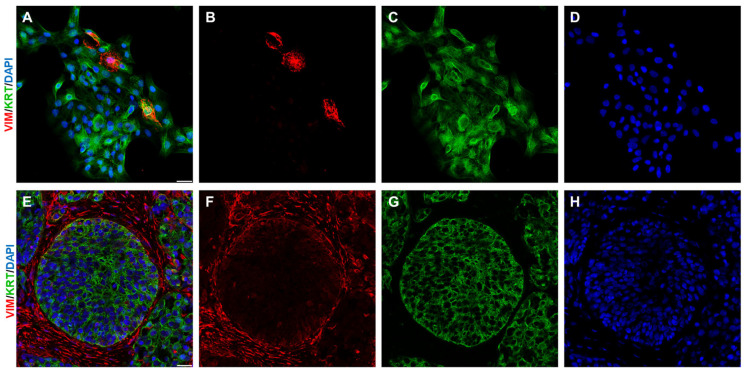
During EMT, epithelial cells gain mesenchymal traits. This shift in phenotype can be visualised by co-expression of epithelial markers (e.g., keratins (KRT) in **C**,**G**) along with mesenchymal markers (e.g., vimentin (VIM) in **B**,**F**). In vitro (**A**–**D**), the epithelial SCC13 squamous cell carcinoma cell line frequently produces vimentin-positive cells (**B**, vimentin stained in red) on the background of keratin positivity (**C**, pan-keratin KRT). In vivo (**E**–**H**, human squamous cell carcinoma tissue), however, such overlap of vimentin (**F**) and pan-keratin (**G**) is only rarely seen. Cell nuclei were counterstained by DAPI (**D**,**H**). The bar in merged images (**A**,**E**) represents 25 mm. The illustration was collated from earlier research listed in Refs. [36,37].

Metastasis is a key characteristic of malignant tumour progression. It is a highly complex process that typically begins with tumour growth, invasion, remodelling of the extracellular matrix, intravasation, and extravasation and ends with the colonisation of distant tissues by cancer cells. A crucial mechanism enabling cancer cells to migrate during metastasis is epithelial–mesenchymal transition (EMT). Colonisation of distant tissue is facilitated by mesenchymal–epithelial transition (MET) [4]. EMT (Figure 1) and MET are opposing processes involved in embryogenesis, regeneration, and metastasis. During EMT, cancer cells gain mesenchymal traits [27]. After undergoing EMT, they lose intercellular adhesions and apico-basal polarity and acquire the ability to migrate and invade, which enables their dissemination to distant tissues where micrometastases form. Metastasis concludes with colonisation, followed by further proliferation of cancer cells, eventually leading to macrometastasis. The course of this process varies depending on the specific type of carcinoma. However, it appears that EMT is not strictly essential for metastasis, as some cancer cells can migrate via amoeboid movement. The main difference between EMT-dependent migration and amoeboid migration is that the latter does not involve enzymatic degradation of the ECM; instead, these cells deform and squeeze through gaps between ECM fibres [28,29]. The exact mechanisms by which tumour cells select their target tissues remain unclear. Nonetheless, factors secreted by cells within the tumour microenvironment seem to contribute to establishing the so-called pre-metastatic niche. This review primarily addresses the issues of the TME. In a historical perspective, Stephen Paget first proposed the famous “seed and soil hypothesis”, highlighting the increased tendency of the stereotypical occurrence of metastasis in certain organs [30]. More recently, the concept of premetastatic niche (PME) was postulated [31] and is noteworthy. The PMN is characterised as a hospitable tissue microenvironment offering a safe harbour for the colonisation of metastatic tumour cells, facilitating their tumour settlement and growth in distant organs. Despite apparent similarities, there is a principal difference. The TME can be gradually crafted by direct contact of cells or via paracrine signalling in situ. To create a PMN, the tumour must act over a long distance; thus, it should already be considered a systemic disease. The exact mechanisms of preferential cancer metastasis to particular body sites remain somewhat elusive, even a century later. Nevertheless, the PMN thus represents a complex microenvironment, with ongoing interplay of numerous cellular and various molecular constituents. Once constituted, the PMN represents a microenvironment with enhanced vascular permeability, prone to angiogenesis, and with notable immunosuppressive and cancer-promoting features [32].

Tumour cells that migrate to form metastasis must be able to survive without a connection to the matrix, since in that situation, many cell types undergo anoikis (a specialised form of apoptosis). Anoikis is an important characteristic of nucleated cells (except leucocytes). For instance, CSCs exhibit resistance to anoikis, which enables their survival within the circulatory system. Multiple underlying mechanisms mediate this unique feature [33,34]. Metastasis represents a critical complication of carcinogenesis, which is often fatal. Therefore, inhibiting metastasis from the primary tumour and inducing its transition into a chronic controlled state offers a promising therapeutic strategy, particularly in patients with inoperable tumours [35].

Metastases are one of the main prognostic markers of HNSCC, mainly spreading to the neck lymph nodes. Neck lymph node metastases reduce survival by 50%. Tumour cells can spread via local invasion and via lymphatic or blood vessels. Metastatic spread in HNSCC is supported by many different mechanisms and interlinked signalling cascades, such as cytokine signalling, involving the activation of TGF-β and CXCR4, which ultimately enhance cell migration and invasion, leading to metastasis. TGF-β can trigger EMT and supports the aggressive phenotype of HNSCC. Moreover, TGF-β controls the activity of genes like cadherin-E or vimentin. These genes are involved in adhesion, movement, and invasion. So the influence of TGF-β can generate tumour cells that are highly mobile and capable of spreading to distant parts of the body [38].

So, what makes metastasis hot, cold, or lethal? There is progressively growing evidence that cancer cells can disseminate from primary lesions much earlier than the classical metastasis models predicted [39]. This is a well known phenomenon in some particular cancer types, namely melanoma. As most morbidity and mortality in solid cancer stems from metastases, it seems critical to prevent the migration of these cells capable of dissemination. This is the theoretical basis of the migrastatic strategy suggested in the last decade [35]. It seems well documented that malignant cells in a mesenchymal-like state can survive using their dormancy programme for extended periods, even years. The mechanisms supporting their dormancy and maintenance of their potential are poorly understood. More recently, it was suggested that, for example, the transcription factor ZFP281 induces transcriptional programmes in disseminated tumour cells that associate with a dormancy signature and phenotype in vivo [40]. Notably, downregulation of ZFP281 in the mouse model leads to a switch to lung metastatic outgrowth. Its human homologue, ZNF281—a zinc-finger transcriptional regulator—has been characterised as an EMT-inducing transcription factor, suggesting its involvement in the regulation of pluripotency, stemness, and cancer [41]. ZNF281 also contributes to organ fibrosis triggered, e.g., by the pro-fibrotic cytokine TGFβ1. Importantly, ZNF281 is also a key regulator of fibroblast activation and myofibroblast differentiation upon fibrotic stimuli. This is a critical step in controlling extracellular matrix composition and remodelling. It seems likely that therapeutic tools regulating these mechanisms of cancer cell dormancy would have a certain therapeutic potential.

If metastasis is not prevented, is there a way to convert its environment into an immunologically “hot” one, with more favourable features for patients? Intralesional interventions for metastases were occasionally used and have proven beneficial in certain patients, even in recent years. Multiple drugs can be injected into metastases, including various chemotherapeutic agents (methotrexate, bleomycin, 5-fluorouracil) [42]. However, due to the immunosuppressive microenvironment established in the metastasis, the release of tumour antigens does not sufficiently elicit systemic immune responses. These procedures are certainly not expected to be curative, but they are frequently associated with favourable effects, e.g., a reduction in the size of the treated and otherwise unresectable metastasis.

Surprisingly, there are well documented benefits of intraleasional administration of drugs which are usually not used in cytotoxic chemotherapy. Among those, rose bengal, a water-soluble xanthine dye historically used in ophthalmology, has more recently been identified as a potential anti-neoplastic agent, particularly in melanoma metastasis treatment [43]. Following intralesional application, tumour cell death stimulates local immune response, and in some patients, it is also associated with tumour necrosis in both adjacent non-injected lesions and occasionally in distant metastases [44]. This suggests a broader effect and involvement of systemic immune response, which was confirmed in studies combining this dye with dendritic cell vaccines [45]. This simple historical dye can also be significantly modified, and loading of rose bengal into vesicle-like constructs of amphiphilic triazine–carbosilane dendrons (dendrimersomes) was tested in the treatment of epidermal carcinomas [46]. This is a remarkable example of drug repurposing and also of a smart redesign of long-known core chemical structures.

More recently, oncolytic viral therapies became another novel category of non-surgical treatment options available [47]. Injected genetically modified viruses (e.g., herpes viruses, coxsackie A21 virus, vaccinia virus) not only induce tumour cell lysis, but their application leads to a remarkable systemic antitumour activity through selective infection of tumour cells. The effect of tumour cell lysis can be further enhanced, e.g., by simultaneous therapy with ICIs. In recent years, there have been numerous oncolytic viral therapies in various stages of development for the treatment of malignant melanoma [48] and other tumours, including SCC [49,50,51]. However, this is still not in routine clinical use; it will be some time before clinicians can engage with viruses used as drugs.

Intriguingly, intratumoral administration of living bacteria can also help ignite an immune response in metastasis. This concept is nothing new, as it has been studied since the nineteenth century [52]. The intralesional application of Mycobacterium bovis (BCG, Bacillus Calmette–Guérin) is a long-known method with some effects in the treatment of several types of tumours [53]. However, intralesional BCG does not usually significantly improve overall survival. Nevertheless, this effect can be enhanced, e.g., by a combination of BCG lysates with thermosensitive hydrogel carriers [53,54]. In patients with metastatic melanoma, intralesional BCG–hydrogel administration was associated with enhanced antigen processing and presentation, maturation of dendritic cells, and a shift towards an M1 phenotype of macrophages and enhanced T cellular responses [55]. This altogether positively correlated with patient survival [53].

Intralesional interventions can also be combined with radiotherapy. Radiotherapy itself can trigger the so-called abscopal effect, where, in some cases, radiotherapy results in a systemic anticancer response clearing even the non-irradiated metastatic lesions at a distance from the primary site of irradiation [56]. The abscopal effect is a rare event in oncology, and it is currently not predictable due to the lack of biomarkers for such a response. Due to the paucity of reliable data, our understanding of the mechanisms eliciting such a response remains vague [57]. There are attempts to combine intralesional therapies with radiation to induce the abscopal effect more regularly. In a mouse model of breast cancer, systemic and abscopal effects were frequently seen when radiotherapy was combined with BCG [58]. This combination seems to reshape the landscape of the immune microenvironment and mitigate leukocyte-like responses by enhancing the infiltration of CD8^+^ T cells, promoting dendritic cell maturation, reducing the infiltration of immunosuppressive cells, and downregulating the expression of immunosuppressive cytokines. Similarly, following radiotherapy, the intratumoural injection of a genetically attenuated strain of Salmonella coated with antigen-adsorbing cationic polymer nanoparticles extended survival in multiple tumour models in mice, including a model of metastasis and recurrence. The antitumour effects were abrogated by the antibody-mediated depletion of CD8^+^ T cells, indicating that adaptive immune responses caused systemic tumour regression [59].

It was suggested based on genome-wide CRISPR screening in mice that SFRP2 is a potential stromal regulator of the abscopal effect of radioimmunotherapy. SFRP2 exhibits CAF expression and radioimmunotherapy-mediated upregulation in unirradiated tumours. Serum proteomics reveals that irradiated tumour-secreted PAI-1 triggers the fate transition of distant tumour pericyte cells into SFRP2-high CAFs via the LRP1/p65 axis. Pharmacologically blocking SFRP2 or PAI-1 enhances the abscopal effect in humanised patient-derived xenograft models [60].

However, this is a good example of making the metastasis immunologically “hot” again. Nevertheless, there is an urgent need to acquire more robust data in humans, where the induction of the abscopal effect remains rare and where its mechanisms remain elusive [61].

## 2. The Tumour Microenvironment and Its Components

**Figure 2:**
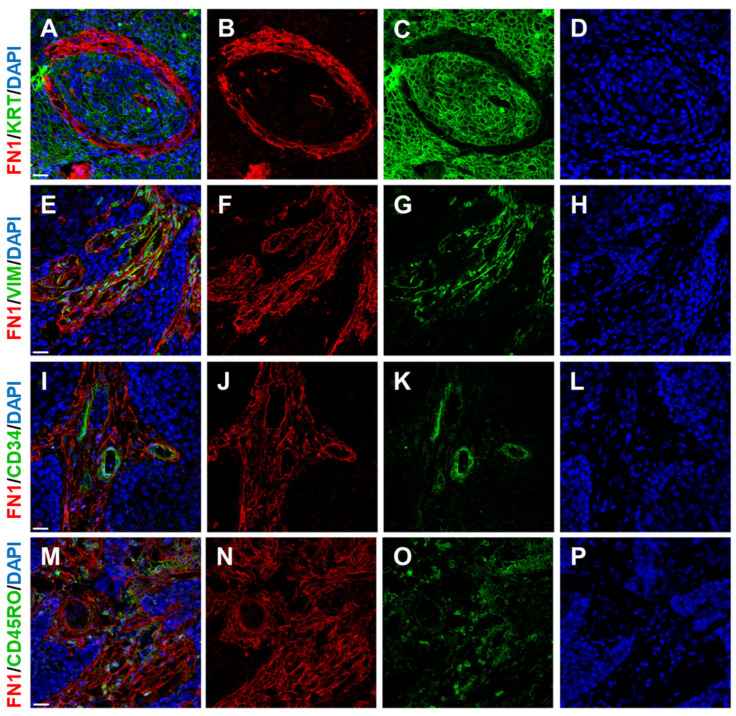
Human squamous cell carcinoma tissue contains several important components, namely malignant epithelial cells and stromal tissue. The extent of tumour stoma can be visualised by, e.g., fibronectin (FN1 in **B**,**F**,**J**,**N**) as a representative of its extracellular matrix. Malignant epithelial cells express various keratins (pan-keratin (KRT) in **C**). The cellular components of tumour stroma mainly represent CAFs expressing vimentin (VIM in **G**). Delicate stromal neovascularisation can be visualised using, e.g., an antibody against the endothelial marker CD34 (CD34 in **K**). Scattered immune cells can be visualised by an antibody against CD45RO, a 180 kDa isoform of CD45 (CD45RO in **O**), which is expressed by most thymocytes, activated memory T cells, granulocytes, and monocytes. Cell nuclei were counterstained by DAPI (**D**,**H**,**L**,**P**). The bar in merged images (**A**,**E**,**I**,**M**) represents 25 mm. The illustration was collated from earlier research listed in Refs. [14,62,63].

The TME comprises cancer cells, including cancer stem cells, alongside immune cells, vasculature, cancer-associated fibroblasts (CAFs), and the bioactive molecules they secrete (Figure 2) [4].

### 2.1. Immune Cells

Immune cells often infiltrate tumours, but they rarely eliminate malignant cells. Tumour cells frequently harbour extensive mutations yet often fail to express recognisable surface antigens, allowing them to evade immune detection. Paradoxically, infiltrating leucocytes often even promote tumour progression. A specific example is tumour-associated macrophages (TAMs). TAMs adopt an M2-like phenotype after being polarised by cells within the tumour ecosystem. They secrete the Interleukins (IL-) IL-4, IL-6, IL-8, and IL-10, TGF-β (Transforming Growth Factor), EGF (Epidermal Growth Factor), and other factors that facilitate tumour growth and cell migration [64]. Myeloid immunosuppressor cells have a similar function [65]. Cytotoxic CD8^+^ T cells in malignant tumours often express the PD-1 receptor on their surface. Tumour cells express a ligand for this receptor (PD-L1). The binding of the ligand to the receptor functionally exhausts CD8^+^ lymphocytes and may induce apoptosis. This process leads to immune surveillance failure, promoting tumour growth, invasiveness, and metastasis. Therefore, the use of immunotherapy to treat advanced HNSCC, particularly through immune checkpoint inhibitors, has proven effective in a subset of patients. It has significantly improved the prognosis of many [66,67]. However, it should be noted that treatment is relevant only in patients where the expression of these molecules has been confirmed. Previous chemotherapy may be helpful in these cases [68]. Additionally, PD-1/PD-L1 blockade is associated with the risk of excessive immune activation, which could lead to adverse effects related to inflammation and autoimmune reactions [69]. Immune cells also play a significant role in the pathogenesis and progression of HNSCC. Although immune cells often infiltrate the tumour stroma (especially cytotoxic T lymphocytes), their anticancer functions are considerably impaired due to the active formation of an immunosuppressive TME. This is usually mediated by cytokines such as IL-10, TGF-β, recruitment of Treg lymphocytes, and MDSCs (myeloid-derived suppressor cells), which hinder effective immune activation [70,71].

In the broadest sense, based on immune activity within the TME, we can divide tumours into two groups. The first group consists of so-called hot tumours, characterised by inflammation. These tumours have a high infiltration of immune cells, over-expression of PD-L1, genomic instability, and pre-existing antitumour immune responses. Hot tumours are usually more responsive to immunotherapy, such as ICIs. The opposite group, “cold” tumours, typically show poor response to immunotherapy. This area is particularly promising and may be especially useful in treating malignant tumours [72].

Nevertheless, the immune infiltrate is much more complex, with striking local intratumoral heterogeneity. This is due to genetic and cellular diversity within the invading immune cells. Importantly, this can be a basis for the failure of immunotherapy and an inferior antitumour immune response [73]. It also reflects an intricate network of intercellular regulations and contraregulations among immune cells. For example, regions occupied by increased numbers of CD206-positive macrophages functionally diminish the functions of present T cells. The resulting suboptimal T cell activity in “cold” tumours persists even after immunotherapy. Mechanistically, this effect is attributed to CX3CL1 produced by the cold tumour cells.

Thus, it seems evident that simple quantification of CD8 T cells alone cannot determine the competence of these cells to attack tumour cells. Instead, it was suggested that activated tumour-specific tissue resident memory CD8 T cells can be defined by the co-expression of CD103, CD39, and CD8 in colorectal cancer [74]. The validity of this concept was supported by clinical data, which showed that patients with low numbers of activated TRM cells had a poor prognosis, even with high CD8^+^ T cell infiltration, and vice versa.

In HNSCCs, combined single-cell RNA sequencing and multiplex immunofluorescence recently identified a new tumour-infiltrating cell (TIL) subtype, CD103^+^ CD8^+^ TILs, associated with clinical response. More specifically, CD103^+^ CD8^+^ TILs were tumour-reactive T cells, and ICIs targeting PD-1 enhanced CD103^+^ CD8^+^ TILs cytotoxicity against tumour growth in vivo [75].

Mutational status should also be considered in immunotherapy outcome prediction. It has been documented that somatic mutation of NFE2L2 leads to Nrf2 activation and radioresistance in HNSCC cells [76]. It is also accompanied by increased recruitment of intratumoral polymorphonuclear myeloid-derived suppressor cells and reduction in M1-polarised macrophages [77]. Interestingly, treatment with the glutaminase inhibitor telaglenastat (CB-839) [78] is capable of overcoming radioresistance. Moreover, it has been hypothesised that it also switches intratumoral myeloid cells to an antitumor phenotype.

Altogether, the tumour ecosystem’s state must be carefully assessed. The overall evaluation appears to benefit from deep molecular profiling and immune phenotyping, and it must be aligned with patient outcome data in the longer term. Of note, approximately 50% of the TME proteome is differentially expressed between cold and hot regions [79].

### 2.2. Cancer Association Fibroblasts (CAFs)

**Figure 3:**
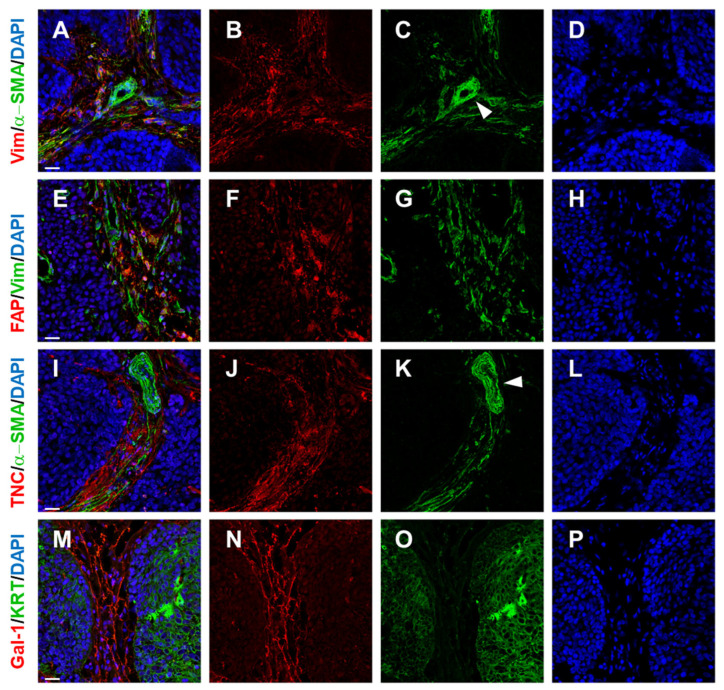
Human squamous cell carcinoma tissue and its CAFs (**A**,**E**) and ECM (**I**,**M**). As typical mesenchymal cells, CAFs express vimentin (Vim in **B**,**G**). This marker is not specific to CAFs. Similarly, α-SMA (αSMA in **C**,**K**) is also not a universal marker for all fibroblasts in tumour stroma; it is co-expressed with vimentin only in a variable proportion of CAFs (depending on, e.g., tumour type). Of note, α-SMA-positive cells are present in the vascular wall of larger stromal vessels (**C**,**K**; vascular structure highlighted by white arrowhead). Similarly, Fibroblast Activation Protein (FAP) is positive in a subset of stromal cells (**F**). The composition of the extracellular matrix in tumour stroma (**I**,**M**) is complex, and the ECM can significantly influence behaviour of malignant cells (pan-keratin KRT staining of malignant cells in **O**). The ECM contains, e.g., Tenascin C (TNC in **J**) and also galectin-1 (Gal-1 in **N**). Cell nuclei were counterstained by DAPI (**D**,**H**,**L**,**P**). The bar in merged images (**A**,**E**,**I**,**M**) represents 25 mm. The illustration was collated from earlier research listed in Refs. [7,63].

Another important component of the TME is CAFs, whose main functions include ECM production, the synthesis of proteases for ECM biodegradation, and the production of various other bioactive cytokines, chemokines, and growth factors. They often also express α-SMA (α-smooth muscle actin), which is a key feature of myofibroblasts, one of the subpopulations of these cells (Figure 3). Unfortunately, there is no specific feature of CAFs. Genes that are expressed differently in CAFs compared to normal fibroblasts have been identified through transcriptional profiling [80]. The origin of CAFs is not uniform. Local fibroblasts or mesenchymal stem cells are likely primary sources. Notably, they express the topologically appropriate HOX code based on their site of origin in embryogenesis [81,82]. However, there is also evidence that they may originate from, for example, pericytes, endothelial cells, and macrophages [14,83].

Pericytes in tissues retain characteristics of progenitor cells [84] and can differentiate into other cells under normal physiological conditions and into myofibroblasts under pathological fibrotic conditions [85]. It has been suggested that cancer-derived exosomes play an essential role in regulating the transition of pericytes to CAFs. Mechanistically, this exosome-mediated transition is achieved via cargo, including Bone Morphogenetic Proteins (BMP) and PI3K/AKT and MEK/ERK pathway activation [86].

Regarding the endothelium, an increasing number of studies have also implicated the endothelial-to-mesenchymal transition (EndMT) as a principal important topic in cardiac development [87], but also as a potentially disease-driving mechanism in pathologic processes, including organ fibrosis [88] and cancer [81].

It has been documented that the EndMT can be a process initiated by activation of endothelial TGF-β signalling. It has been suggested that it leads to a further increase in TGF-β signalling, thus establishing a positive feedback loop with the EndMT, leading to more EndMT [89]. Such self-intensifying vicious circuits are particularly interesting with respect to cancer progression. Notably, metabolic reprogramming of endothelial cells within the tumour microenvironment also contributes to the EndMT. This can be explained by metabolic modulation. In detail, atypical production of acetate from glucose results in acetylation of the TGF-β receptor ALK5 and SMAD2 and SMAD4, leading to activation and long-term stabilisation of TGF-β signalling [90]. CAFs are potent producers of multiple cytokines in the TME, including TGFb [91]. Besides this, it has been reported that CAF-derived PAI-1 also promotes the EndoMT in cervical SCC. This can also initiate tumour neolymphangiogenesis, which facilitates cancer cell intravasation/extravasation, which in turn promotes lymphatic metastasis [92].

The hypothesis that CAFs may derive from mesenchymal cells is supported by the fact that CAFs in basal cell carcinomas can further differentiate into adipocytes, osteoblasts, or chondrocytes. This suggests that CAFs are multipotent cells, similar to mesenchymal stem cells [93]. In vitro experiments and animal models have indicated that CAFs can also originate from tumour cells through the epithelial-to-mesenchymal transition (EMT) [94,95]. The primary driver of CAF formation from their precursors is TGF-β, with the contribution of Gal-1 [96].

CAFs are not a homogeneous family; rather, they constitute a broad and functionally diverse group. Myofibroblastic CAFs (myCAFs) express α-SMA and actively produce ECM. Occasionally, cells that generate ECM are called matrix-producing CAFs (matCAFs). There is no strict boundary between myCAFs and matCAFs. Fibroblasts known as inflammatory CAFs (iCAFs) promote inflammation, while antigen-presenting CAFs (apCAFs) are active antigen-presenting cells. A subpopulation of CAFs, characterised by high expression of ID genes, is responsive to TGF-β and potentially serves as a precursor for other CAF subpopulations [97,98,99]. CAFs exert a significant paracrine influence on surrounding cells, both normal and tumour cells. CAFs isolated from cutaneous basal cell carcinoma induce keratins 8 and 19, vimentin (a marker of mesenchymal cells), and transcription factor snail in normal keratinocytes in vitro, indicating the low differentiation and EMT of these keratinocytes. Similarly, cancer cells (HNSCC, melanoma) cocultured with normal fibroblasts alter their phenotype to CAFs [3]. However, fibroblast stimulation in this context is usually temporary [100], often explained by epigenetic modifications such as DNA methylation [101]. Some mediators involved in communication between tumour cells and CAFs—such as BMP-4, IGF-2, IL-6, IL-8, CXCL-1, and TGF-β—may be potential therapeutic targets [14]. The impact of CAFs on tumour cells appears to be universal. For instance, CAFs from melanoma not only influence its tumour cells and keratinocytes but may also affect cells from other tumour types, such as glioblastoma or breast cancer. This could hold significance for cancer therapies targeting tumour stroma [37,102].

As already mentioned above, CAFs produce the extracellular matrix (ECM) of the tumour. In addition to commonly found molecules such as collagens and fibronectin, the production of tenascin-C is also important and can be found in both the tumour stroma and the healing wound. Among other things, tenascin-C is responsible for stimulating aggressive growth in many types of tumours [103,104]. The most robust ECM production has been observed in ductal adenocarcinoma of the pancreas, which has a desmoplastic character [105]. This fact may contribute to the high therapeutic resistance of this tumour [106]. Generally, a high number of matCAFs is a sign of a very poor prognosis, no matter the specific type of tumour. Differences in ECM production in comparison to normal tissues influence the mechanical characteristics of cancer tissue, which may also lead to changes in the biological characteristics of the tumour [107].

Collagens are the most structural ECM components of normal and tumour tissues, including HNSCCs [108]. However, CAF-deposited collagen undergoes progressive fibre reorganisation and enzymatic cross-linking in the inflammatory microenvironment [109]. This correlates with tissue stiffening, increased tumour cell invasion, and poor survival [110].

The linearization of collagen can be microscopically visualised as an overall increase in tissue birefringence. Structural patterns of fibrillar collagen organisation are termed “Tumour-Associated Collagen Signatures” and have been used to classify the changes in collagen arrangement that accompany carcinoma progression [109]. This progression is usually most striking at the invasive front of the tumour, where the stiffness of the stroma and cellular mechanosignaling is highest [111]. Intriguingly, the most significant number of infiltrating macrophages and the highest level of TGFβ signalling were found within cells at the invasive front. It seems that the difference in stiffness can also be key to immune infiltration and response [112]. Stiffness can be one of the limiting factors for the access of immune cells to the tumour [113,114], and collagen and collagen degradation products regulate the phenotypes and functions of immune cells via, e.g., collagen-binding endocytotic receptors Endo180 and mannose receptor (CD206) on macrophages [115].

Fibronectin is a glycoprotein with a multitude of functions in normal tissue and in the tumour stroma. It is not a cancer-specific ECM. However, it has been noted that the extracellular matrix protein fibronectin contains a domain that is rarely found in healthy adults and is almost exclusively expressed by newly formed blood vessels in tumours [116]. Similarly, O-glycosylated oncofoetal fibronectin is found in foetal and cancer cells, but usually not in healthy tissues.

It seems that it is not easy to predict the role of fibronecin in tumours [117]. Using in vitro models of HNSCC, it was documented that the ECM type plays a major role in HNSCC cell phenotype, influencing proliferation, migration, and invasion [118]. However, the observed effects of ECMs were cell line-dependent. Fibronectin harbours three repeating units, named type I, II, and III repeats, that contain various binding sites for other molecules, e.g., collagen/gelatin, integrins, and heparin. Polymerisation and pericellular fibronectin assembly associate fibronectin dimers into fibrils, and cell-binding activity enables these fibrils to participate in various physiological and pathological functionalities [119]. As a provisional matrix protein, fibronectin assembly provides a template for the assembly of collagen and integration of other proteins into the tumour ECM, including matrix components involved in TGFβ signalling [119]. In HNSCC, stromal over-expression of fibronectin, determined by immunohistochemistry (Figure 2), was shown to be an independent unfavourable prognostic indicator of overall and disease-free survival [120].

It has been suggested that, in HNSCC, the oncofoetal form of fibronectin is a major and obligate component of the matrix assembled by stromal fibroblasts. In contrast to earlier views, fibronectin was more recently determined to be an independent unfavourable prognostic marker in HNSCC and other cancer types [121]. Mechanistically, cancer cells readily migrate onto fibrillar fibronectin-rich matrices, which is achieved through αvβ6 and α9β1 integrin interaction [120].

Interestingly, the oncofoetal form of fibronectin was demonstrated to be specifically associated with the M2 polarisation of intratumoural macrophages [122]. It has also been suggested that upregulation of the O-glycosylated oncofoetal fibronectin can be linked to the acquisition of a multidrug resistance phenotype in a breast cancer model exposed to chemotherapy [123]. These unusual extracellular matrix glycoproteins can be used as potential therapeutic targets for the treatment of cancer. This underscores the importance of tumour–stroma interaction in the orchestration of the collective mode of migration in HNSCC [118].

Tenascin C is another large matrix glycoprotein with several alternatively spliced forms and binding partners. Tenascin-C protein synthesis is tightly regulated, with widespread protein distribution in embryonic tissues but restricted distribution of tenascin-C in adult tissues. Tenascin-C is also expressed de novo in adults during wound healing [124], inflammatory disorders [125], or in tissue fibrosis [126]. Similar to oncofoetal fibronectin, tenascin C expression is intensely studied in pathologies, including cancer. In HNSCC, intense staining of tenascin C is observed in the tumour stroma (Figure 3), with pronounced immunoreactivity frequently surrounding advancing tumour cell islets [104]. This implies a possible role of Tenascin C in cancer invasion. In a rare genodermatosis known as recessive dystrophic epidermolysis bullosa, a severe genetic disease caused by mutations in the collagen COL7A1 gene, it is associated with fibrotic scarring of the skin, pseudosyndactyly, and an early onset of frequently fatal cutaneous squamous cell carcinoma. In a mouse model post-injury, persistent dermal fibrosis with elevated levels of Collagen I, α-SMA^+^ myofibroblasts, and Tenascin-C was observed [127]. This altogether makes an excellent environment for highly invasive and frequently fatal SCC [128]. Notably, tenascin C was recently shown to facilitate tumour progression in a carcinogen-induced immunocompetent murine model of oral squamous cell carcinoma by promoting an immune-suppressive stroma [129]. Gal-1 belongs to the family of carbohydrate-binding proteins with a high affinity for β-galactose-containing oligosaccharides [8]. Gal-1 is expressed in many sites under normal and pathological conditions, including SCC (Figure 3). Similar to previous ECM molecules, Gal-1 exerts a timely, precise modulatory role in different stages of wound healing. Conversely, persistent over-expression of Gal-1 enhances angiogenesis and ECM production, leading to keloid scar formation and organ fibrosis, respectively. In tumour stroma, galectin-1 is an essential player in the induction of myCAFs from resident fibroblasts; this effect seems to be TGFβ-independent [96]. Galectins, as a family, are versatile proteins involved in immunomodulation. Evidence suggests that galectins can control the immunoregulatory function of cytokines and chemokines through direct binding. There is evidence that galectin-1 binds, e.g., C-X-C motive chemokine ligand 4 (CXCL4). Regarding immunomodulation, CXCL4 significantly increases the apoptotic activity of Gal-1 on activated CD8^+^ T cells, while no effect is observed in CD4^+^ T cells [130]. Of note, Gal-9/CCL5 (Chemokine (C-C motif) ligand 5) heterodimer significantly reduces the Gal-9-induced apoptosis of CD4^+^ T cells and not of CD8^+^ T cells. This underscores specific galectin–chemokine interactions and the versatile immunoregulatory function of galectins. In HNSCC, Gal-1 was linked to poor prognosis [63]. Similarly, in gastric cancer, it was suggested that heparanase and Gal-1, which could regulate ECM remodelling and tumour immune microenvironment, contribute to 5-fluorouracil chemoresistance [131].

In conclusion, CAFs play a critical role in HNSCC pathogenesis (e.g., formation of ECM, angiogenesis, and immune and metabolic reprogramming of the TME, impacting metastasis and therapeutic resistance). They possess many molecules and signalling pathways that could be important targets in future oncological therapy [132]. On the other hand, production of CXCL14 in iCAFs is related to better prognosis [98]. Production of inflammation-stimulating cytokines is connected with the change in cell metabolism influenced by hypoxic conditions in tumour stroma and related to the expression of FAP (Fibroblast Activation Protein) in these cells [133].

So, what makes CAFs hot, cold, or lethal? Accumulating evidence has demonstrated that the overall proportion, phenotype, and distribution of CAFs within the tumour microenvironment are important in determining responses to ICIs [134]. This functional clustering of CAFs seems to be a rather universal phenomenon, as evidenced across several types of tumour, including HNSCC [135]. Sadly, heterogeneity in the cellular composition of tumours in individual patients represents a major challenge in modern oncology. CAFs, in the broad sense of the term, have been reported to promote the establishment of an immunologically cold TME [136]. CAFs act both through direct modulation of immune cell phenotypes and indirectly via inhibition of immune cell recruitment and infiltration into the developing tumour [137]. Of note, CAFs of clusters characterised by extracellular matrix proteins and TGFβ signalling are indicative of primary resistance to immunotherapies [134]. In detail, Kieffer and co-workers demonstrated that the abundance of ECM-producing CAFs and TGFβ-regulated CAFs in tumour samples is anticorrelated with CD8^+^ T cell infiltration but correlated with PD-1^+^ and CTLA4^+^ CD4^+^ T cell content in breast cancer. Moreover, these observations imply the existence of a reciprocal crosstalk between these two CAF clusters and Tregs. TGFβ-regulated CAFs drive T cell exclusion, preventing intratumoral T cells from engaging cancer cells [138]. Collectively, this suggests the development of novel tools for the therapeutic targeting of a specific CAF subpopulation, aiming to enhance clinical responses to existing immunotherapy.

### 2.3. Blood Vessels

An important part of the TME is also blood vessels, which are formed inside the tumour stroma by a process known as neovascularisation. Neovascularisation and the abundant tumour permeation by capillaries supply nutrients to the tumour. Specifically, targeting new vessels in cancer treatment is one of the most frequently used strategies [14]. Despite abundant neovascularisation, hypoxic areas can be found in solid tumours. These areas are not typically present in healthy tissue. Hypoxia itself promotes resistance of tumour cells to chemotherapy and radiotherapy and promotes tumour progression overall [139].

Overall, low pO2 (partial oxygen pressure) in the tumour microenvironment prior to initiation of therapy indicates a worse prognosis for patients [140]. Some studies suggest that tumour hypoxia is partially related to the amount of haemoglobin in the blood, and thus, tumours in anaemic patients will have lower pO2. Nonetheless, both well and poorly oxygenated tumours can be found in patients with normal haemoglobin levels [141]. Both tumour and nontumour cells in the tumour ecosystem produce factors such as HIF, VEGFA (Vascular Endothelial Growth Factor), IL-6, and IL-8, which stimulate endothelial cell proliferation and thus tumour vascularisation [142,143]. Notably, in HNSCCs, cancer stem cells are localised in perivascular niches and rely on crosstalk with endothelial cells for survival and self-renewal. It was suggested [144] that IL-6 levels in tumour-associated endothelial cells correlate with the tumourigenicity of CSCs, as evidenced in vitro by the p-STAT3 activation, survival, and self-renewal of human CSCs.

There is also evidence acquired in glioblastoma suggesting that hypoxic niches attract and sequester myeloid and cytotoxic T lymphocytes, where they were reprogrammed toward an immunosuppressive state. Mechanistically, it was suggested that chemokine CCL8 and cytokine IL-1β are two hypoxic-niche factors critical for immunosupresive tuning in the tumour mass [145]. On the other hand, the defective wall of tumour capillaries could be exploited for the targeted accumulation of polymeric drugs in the tumour [146].

Tumours are intrinsically heterogeneous, as stated above. The origin of such remarkable cell state diversity remains poorly understood. It was suggested earlier that clinically relevant phenomena such as primary tumour growth and its consequent metastatic dissemination result from distinct cancer cell subpopulations [147]. It was suggested that phenotypic competencies of various tumour cells can be dynamically acquired after exposure to specific niche signals. These cues are associated with a spatially localised perivascular niche. In this context, the phenotype of cancer cells can be acquired through an intercellular communication pathway established by, e.g., endothelial cells. However, this niche is also a shelter for tumour-associated macrophages, which can facilitate cancer progression. In a murine breast cancer model, lymphatic vessel endothelial hyaluronan receptor-1 (LYVE-1)-expressing TAMs form coordinated multicellular nests. In response to IL-6, these TAMs express the immune-suppressive enzyme heme oxygenase-1. Blocking the development of LYVE-1^+^ TAMs in the mouse model resulted in an increase in CD8^+^ T cell recruitment to the tumour and enhanced response to chemotherapy [148].

Lastly, there is also a specific subset of CAFs residing in the perivascular niche [134,149]. These CAFs seem to be responsible for the restriction of T cell extravasation and, consequently, also for tumour immune exclusion. In solid tumours, desmoplasia in tumour stroma represents a formidable challenge to immunotherapies and also to endogenous or adoptively transferred T cells. Depletion of FAP^+^ CAFs in the perivascular niche results in the loss of the structural integrity of the desmoplastic matrix. This reduces myeloid cell accumulation and increases endogenous CD8^+^ T cell and NK cell infiltration. This makes tumours more susceptible to consequent anti-PD-1 antibody-based therapy [150].

## 3. Intercellular Signalling in the Tumour Ecosystem

In the context of tumourigenesis, cells within the tumour ecosystem produce cytokines, chemokines, growth factors, and other molecules, such as TGF-β, monocyte chemoattractant protein 1, IL-6 and IL-8, Tumour Necrosis Factor TNF-β, CXCL-1, and VEGF. They promote tumour-stimulating inflammation, tumour cell proliferation and migration, immunosuppression, and metastasis [142].

**Figure 4:**
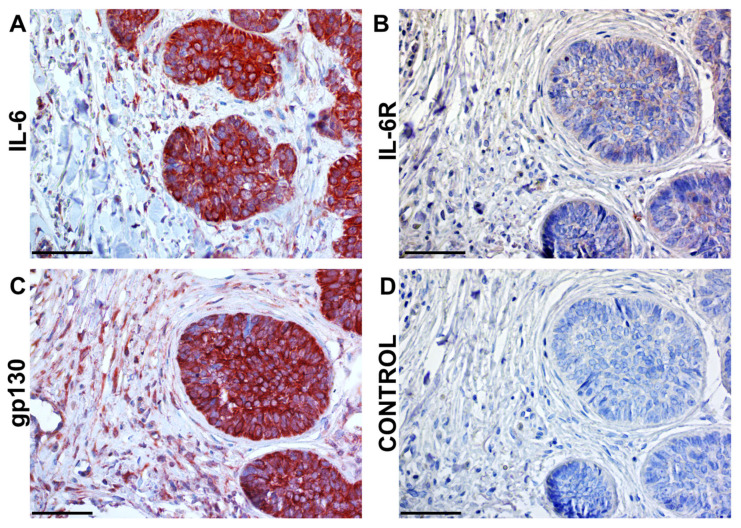
Human squamous cell carcinoma tissue and principal components of the IL-6 signalling cascade. The interleukin IL-6 itself (**A**) is abundantly produced by cancer cells, as well as in the stroma. However, IL-6R (**B**) is predominantly detected in the tumour parenchyma. The signal transducer gp130 (**C**) is broadly present in tumour cells and in stromal populations. Isotype control (**D**) was used to confirm the specificity of the immunohistochemical reaction using AEC substrate (red). Sections **A**–**D** were counterstained lightly by Gill’s haematoxillin. The bar represents 100 mm. The illustration was collated from earlier research listed in Ref. [71].

Specifically, IL-6 is a crucial component of the TME (Figure 4) and can be produced by tumour cells themselves or possibly by CAFs and TAMs. A rise in serum IL-6 levels is generally associated with processes such as inflammation, ageing, tumourigenesis, autoimmune disorders, and psychiatric disorders [71]. With ageing, IL-6 levels increase in tissues and the body, thereby promoting the development of chronic inflammation in the elderly. In any case, ageing-associated chronic inflammation is considered one of the leading causes of ageing-related diseases such as neurodegenerative processes, cardiovascular disease, and carcinogenesis [151]. IL-6 has two different modes of action, either through membrane-bound IL-6 receptors or soluble IL-6 receptors. Transmembrane IL-6Rs are associated with the membrane and form complexes with gp130 (Glycoprotein 130). Upon IL-6 binding, IL-6R tyrosine kinase is activated, and STAT3 is stimulated. Transmembrane IL-6Rs are thus involved in anti-inflammatory and pro-carcinogenic signalling. Soluble IL-6R binds IL-6 in the extracellular environment, and subsequently, the whole complex binds to gp130, which is anchored in the membrane (Figure S1). Soluble IL-6Rs promote inflammatory processes [152].

As described above, the IL-6 signalling cascade offers several candidate targets for therapeutic interventions. In principle, neutralisation of cytokines (e.g., TNFa) via specific antibodies has proved to be a successful strategy in the management of numerous inflammatory and autoimmune conditions [153].

During inflammation, IL-6 levels in the circulation rise dramatically from virtually undetectable in healthy individuals to several-fold higher levels in patients who develop fatal sepsis [154]. Therefore, IL-6 inhibition has also emerged as an attractive therapeutic strategy for translation to the clinic. In principle, the inhibition of IL-6 signalling can be achieved by the application of neutralising antibodies.

However, IL-6 is produced in large quantities by numerous cell types (as evident in Figure 4), and thus, the consumption of neutralising antibodies seems to be a significant hurdle for this strategy. Nevertheless, e.g., the chimeric anti-IL-6 monoclonal antibody siltuximab was approved for patients with idiopathic multicentric Castleman disease [155]. However, the production of IL-6 can be achieved with ease by various anti-inflammatory drugs, including corticoids. This strategy was reported to have some benefits in the management of patients during the COVID-19 pandemic [156,157]. Similarly, the broad expression of gp-130 is high across tissues. Additionally, because many IL-6 family cytokines generally utilise gp130 and gp130 knockout mice are not viable, this increased scepticism towards and hindered the clinical development of gp130 antibodies.

However, small-molecule inhibitors targeting JAKs (Janus Activated Kinases), which act downstream of gp130, represent another FDA-approved approach for JAK/STAT inhibition. Such molecules have recently been used in various indications in the clinic to treat rheumatoid arthritis and other inflammatory diseases [158]. Unfortunately, these JAKs are not exclusively activated by IL-6, so targeting these signalling molecules does not offer selective inhibition of IL-6.

On the contrary, the humanised monoclonal antibodies to IL-6R have reached clinical use. These antibodies prevent the binding of IL-6 to IL-6R. Tocilizumab and sarilumab were approved for the treatment of rheumatoid arthritis [155].

In cancer, the relative scarcity of IL-6 receptors seems to be critical for the precise regulation of the signalling cascade. The amount of IL-6R present at the cell surface determines the responsiveness of the cell to the cytokine and might therefore be decisive in the development of an inflammatory response. It has been shown that IL-6R and gp130 are constitutively internalised independent of IL-6 [159]. This makes the IL-6 receptor, theoretically, a particularly suitable therapeutic target. However, IL-6 receptor blockade has not yielded the anticipated substantial results as an oncological therapy of solid tumours or haematological malignancies [160].

Therefore, new strategies for IL-6 receptor inhibition are in development. For instance, peptides derived from the ABD fragment of streptococcal G protein appear capable of inhibiting both the proliferation and migration of pancreatic ductal adenocarcinoma and melanoma cells [161].

IL-8 is another potent CXC chemokine family member. Following its binding to the receptor, IL-8 (also known as CXCL8) promotes the proliferation, survival, and metastasis of cancer cells and tumour angiogenesis [162]. Together with other chemokines (small chemotactic cytokines), IL-8 shares two types of receptors, CXCR1 and CXCR2. CXCR2 is expressed on a broad spectrum of carcinomas. In SCCs, the expression of both IL-8 and CXCR2 in ESCC was an independent predictor of prognosis [163].

Dual targeting can frequently offer significant benefits in oncology. In experiments where IL-6 and IL-8 were inhibited in melanomas, these interleukins were found to be essential for promoting the activity of CAFs. When IL-6 and IL-8 are deficient in the environment, the invasiveness of melanoma cells is suppressed [164,165].

For example, therapeutic blockade of IL-6 and IL-8 in mice using Tocilizumab (a humanised monoclonal antibody against the α chain of IL-6) and Reparixin (a low-molecular-weight inhibitor of IL-8 signalling) seems to be very effective in suppressing breast cancer metastasis in experiments. Recently, blockade of the IL-6 and IL-8 receptors (IL-6R and IL-8R) with a novel bispecific antibody significantly reduced metastatic burden in multiple preclinical mouse models of cancer [166]. In head and neck squamous cell carcinomas, inhibiting the triple combination of IL-6, IL-8, and CXCL-1 has been shown to be highly effective in reducing tumour cell activity [167].

Another example is MIF (macrophage migration inhibitory factor), which is one of the main pro-inflammatory cytokines. It is produced by several cells and acts as an inhibitor of macrophage migration. At the same time, it promotes macrophage activation, stimulates NO and cytokine production (such as TNF-α, IL-1, and IL-6), and influences cell proliferation and differentiation, which may also contribute to tumourigenesis and metastasis. Evidence of its role in tumourigenesis is shown by the increased MIF mRNA expression found in lymph node metastases from prostate cancer [168]. Nevertheless, it has both positive and negative effects on healing, and its cellular specificity is not fully understood [169]. Furthermore, it also plays an important role as a key enhancer of the effect of oestrogen on wound healing [170]. Oestrogens themselves are crucial regulators of wound repair and healing, especially in the skin. This has been demonstrated through a series of experiments in mice, where the absence of oestrogens led to a significant delay in the healing and repair of skin wounds. More specifically, the contractility of the emerging scar was affected [171].

Macrophage colony-stimulating factor (M-CSF) has become a potentially important factor for targeted cancer therapy. This cytokine is crucial for the differentiation of the monocytic cell line and for encouraging the formation of dense vascular plexuses within tumours. In experiments on murine osteosarcomas, inhibition of M-CSF led to suppression of tumour angiogenesis and lymphangiogenesis [172].

The mentioned systemic therapy using inhibitors of inflammatory molecules causes many side effects. In particular, it adversely affects wound healing, causes immunomodulation, and may lead to further cancer development [170]. Additionally, the ability of tumour cells to develop resistance to oncological drugs plays a significant role. Several preclinical studies have demonstrated that this ability is linked to the activation of cancer-associated NF-κb (Nuclear Factor kappa-light-chain-enhancer of activated B cells). NF-κB inhibits cell death and promotes cell proliferation, angiogenesis, and migration, which likely counteract the cytotoxic effects of chemotherapy and radiotherapy, thereby facilitating resistance development [173]. STAT3 has also been associated with poor responses to cancer therapy. Experimental inhibition of NF-κB and STAT3 in prostate cancer resulted in tumour growth arrest and prevented cancer stem cells from thriving [174]. However, long-term inhibition caused adverse effects such as neutrophilia, liver damage, and acute inflammation, enhanced by IL-1 [175]. IL-1 is crucial for promoting healing by protecting wounds from bacterial infection; nonetheless, its inhibition does not diminish new tissue production [176].

CAFs also produce various factors that influence angiogenesis, including VEGFA, TGF-β, HGF, EGF, and FGFs (Fibroblast Growth Factors). One of the most important factors is VEGFA, which is upregulated in most tumours and plays an indispensable role in their stroma. CAFs also express several receptors, such as Platelet-Derived Growth Factor receptor PDGFR-a and PDGFR-b [177]. They also produce ECM components and enzymes that remodel the ECM, such as NG2 (Neuron-Glial antigen 2), tenascin-C, type I collagen, fibronectin, and matrix metalloproteinases. Nowadays, it is evident that CAFs have an impact on the fate of tumours. The main mechanism of their action is precisely their strong paracrine effect on surrounding cells. The effect of CAFs on tumour cells is to stimulate their proliferation, migration, invasiveness, and EMT, thereby giving tumour cells a stem cell phenotype [178].

Endogenous lectins are proteins distinct from immunoglobulins that specifically recognise saccharide motifs. For example, galectins are highly significant in carcinogenesis and exert complex biological effects, one of their primary functions being the ability to interpret the structure of beta-galactosides [179]. About 15 different galectins have been identified in mammals, 12 of which are present in human cells. Some galectins are broadly distributed across most tissues, while others display more restricted tissue specificity [180]. Notably, they are abundantly expressed in the microenvironment of tumour cells and the granulation tissue during wound healing [181]. Gal-1 and Gal-3 are the most extensively studied galectins in cancer research. Broadly speaking, Galectins 2-15 are involved in tumourigenesis, although their precise roles remain uncertain. Gal-1 expression is regulated by HIF1, which is crucial in shaping the tumour microenvironment [182]. Specifically, upregulation of Gal-1 can significantly influence tumour progression by disrupting cell transformation, proliferation, angiogenesis, cell adhesion, invasiveness, and immunosuppression. Gal-1 is linked to all cancer stages, affecting both tumour cells and stromal cells within the TME [183]. The immunosuppressive effects of Gal-1 involve inhibiting cytotoxic T lymphocytes through binding to their surface via CD4, CD7, CD43, and CD5 [184]. In HNSCC, Gal-1 expression correlates with the presence of αSMA-positive CAFs and is also associated with the expression of several molecules, such as MAP3K2 (Mitogen-Activated Protein Kinase Kinase Kinase 2), TRIM23, PTPLAD1, FUSIP1 (Fused in Sarcoma protein), SLC25A40 (Solute Carrier Family 25 Member 40), Spindlin 1 (SPIN1), and others [63]. Conversely, the interaction of Gal-1 with integrin receptors induces anoikis in tumour cells [185]. Gal-3, produced by the tumour microenvironment, primarily supports cells, including mesenchymal stromal cells. It functions as a regulator of diverse processes crucial for tumourigenesis, such as apoptosis, metastasis, immune surveillance, mRNA splicing, gene expression, and inflammatory responses [186].

## 4. Similarity Between Cancer and Wound Healing

**Figure 5:**
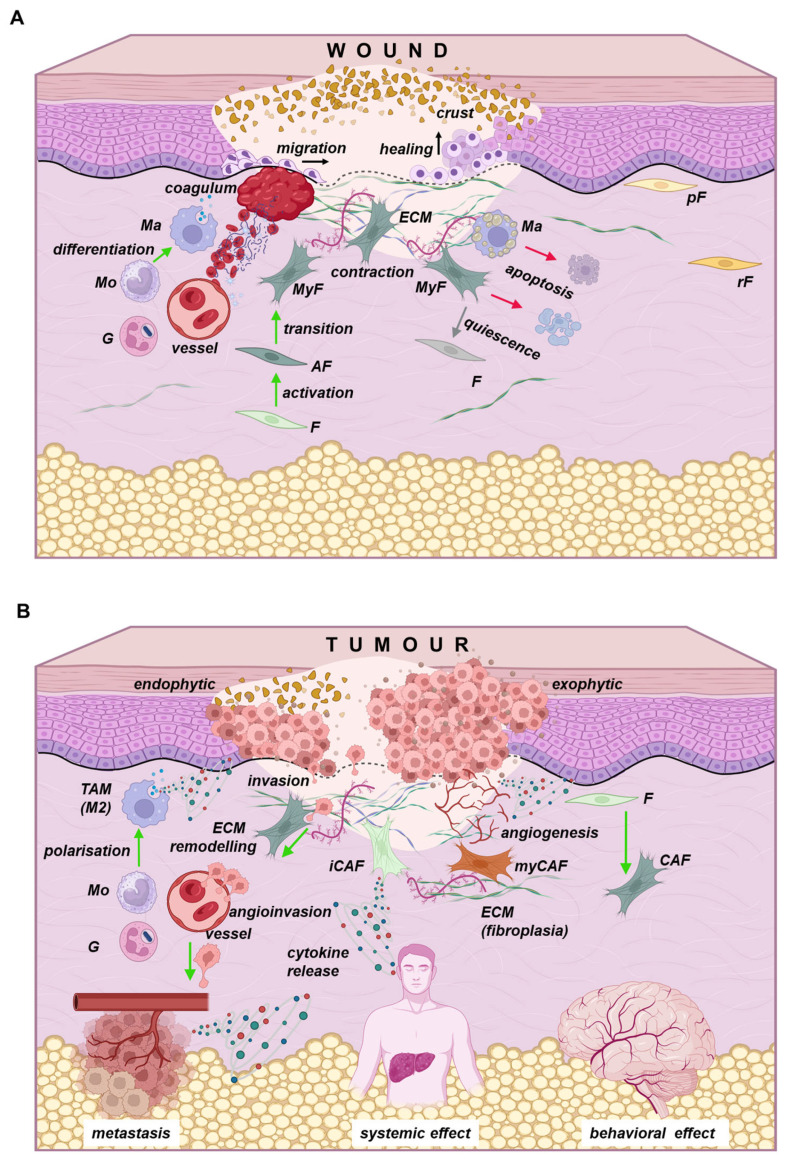
Wound healing (**A**) and tumour growth (**B**) share several similar mechanisms. After a traumatic injury (**A**), a blood coagulum forms, providing a primary seal for the wound site, with dried proteins creating a surface crust. Various immune cells released from the blood, such as granulocytes (G) and monocytes (Mo), which differentiate into macrophages (Ma), contribute to immune surveillance and the removal of tissue debris, including dead cells and extracellular matrix (ECM). Tissues contain different subsets of resident fibroblasts, such as papillary dermis fibroblasts (pF) and reticular dermis fibroblasts (rF). These fibroblasts are generally quiescent cells. Following an injury, local fibroblasts increase their transcriptional and metabolic activity, turning into activated fibroblasts (aF). Eventually, these fibroblasts can transition into myofibroblasts (MyF), which are capable of wound contraction. Once the granulation tissue adequately fills the dermal defect, epithelial cells at the wound’s edge can migrate over the granulation tissue, proliferate to heal the epithelial defect, and reconstitute the epithelial barrier through stratification and differentiation. The tissue then enters a stage of slow remodelling. However, successful healing also initiates negative feedback that reduces overall tissue activity. Unnecessary cells are eliminated by apoptosis or enter a quiescent state. In tumours (**B**), a similar activation of several cell types occurs; however, this activation is sustained and lacks the negative feedback typical of healing. Subsets of immune cells, including granulocytes (G) and monocytes (Mo), are generally present, but this immune response is ineffective at eliminating malignant cells. Moreover, the phenotype of macrophages shifts towards M2 polarised tumour-associated macrophages (TAMs). Resident fibroblasts are recruited through the paracrine release of various cytokines, transforming into cancer-associated fibroblasts of several categories, such as myofibroblastic CAFs (myCAFs) and pro-inflammatory CAFs (iCAFs). Myofibroblasts can secrete ECM, creating a tumour-promoting niche, which may lead to fibroplasia in some tumours. Conversely, CAFs can also degrade the ECM, making it more permissive for invasion and facilitating the invasion of tumour cells. This process allows tumour cells to traverse the vascular wall and enter circulation, leading to the formation of distant metastases. Primary tumours, along with their microenvironments, release various cytokines. These molecules can act locally through autocrine and paracrine effects; however, they can also have long-distance effects, contributing to the formation of a premetastatic niche and resulting in systemic effects in organs such as the liver, adipose tissue, or brain. These systemic effects can lead to conditions like terminal cachexia and depression. Created in https://BioRender.com (accessed on 19 August 2025).

Several studies indicate similarities between cancer and wound healing (Figure 5). These similarities have been outlined since the 1990s [187]. Specifically, we note the resemblance between the granulation tissue formed during wound healing and the tumour stroma [188,189]. Fibroblasts play a key role in both processes; their activation leads to increased proliferation and enhanced productivity. Indeed, activated fibroblasts produce various growth factors, cytokines, chemotactic molecules, and ECM proteins, supporting the formation of both granulation tissue and tumour stroma [190]. Fibroblasts can also differentiate into myofibroblasts, with the expression of αSMA being a major feature of this change. In a healing wound, myofibroblasts facilitate contraction, thereby reducing the area for re-epithelialisation [191].

Fibroblast-activating protein alpha (FAP-α) and dipeptidyl peptidase IV (DPPIV) are located on the plasma membrane of fibroblasts in abnormally healing wounds or on tumour cell surfaces, where both promote cell invasiveness, tumour growth, and keloid scar formation [188]. It should be noted that FAP-α is not present on normal, healthy adult tissue cells. Therefore, inhibiting FAP-α or DPPIV with antibodies (e.g., sibrotuzumab) is considered a treatment option in oncology. However, studies in small-cell lung cancer and colorectal cancer have yielded disappointing results [178].

Healing is a strictly regulated process that involves four basic phases: hemocoagulation, inflammation, proliferation, and maturation/remodelling [71,192]. At the time of injury, cells at the wound border are activated, and there is stimulation of local migration, proliferation, and differentiation of various cell types. Inflammation plays a vital role in this process, serving as protection against infection and aiding in the clearance of tissue debris [189]. This inflammatory response occurs in both healing wounds and tumour stroma. In healing wounds, inflammation gradually resolves, but in tumours, it does not, and the tumour continues to proliferate [193]. Because of this, tumours are sometimes described as “*wounds that never heal*” [187]. The subsequent phase is proliferation, characterised by the formation of granulation tissue, which closely resembles tumour stroma in architecture. Within granulation tissue, fibroblasts produce cytokines, chemokines, and growth factors that stimulate angiogenesis and facilitate re-epithelialisation. Both granulation tissue and tumour stroma can encourage poorly differentiated epithelial cells to proliferate. From this perspective, the tumour can be seen as an unending healing process with significant inflammation [157]. Unlike wound healing, platelets—though essential for healing—are not involved in the tumour stroma of solid tumours [71]. Therefore, healing is a tightly regulated process, and its disruption can lead to abnormal outcomes such as hypertrophic or keloid scars [194].

Cells that support healing and the migration of other cells are currently a very intriguing topic. For example, a recent study on tadpole tail regeneration in the *Xenopus laevis* model used single-cell and spatial transcriptomics to identify a new population of cells that appear only in the early stages of regeneration. These are known as RICs (regeneration-initiating cells). Soon after amputation, RICs emerge at the wound edge and trigger various signalling cascades that regulate the production and breakdown of collagen, laminin, midkine, and thrombospondin. Their activity generally entails remodelling the surrounding ECM and encouraging the migration of cells vital for regeneration. The role of similar supporting cells in the initiation and spread of tumourigenesis in vertebrates is highly likely [189].

So, what are the hot, cold, and lethal aspects of this wound/cancer parallel? Inflammation is an integral part of the healing process. However, if inflammation exceeds the expected duration for any reason, it can result in poor healing [195]. Bacterial populations, especially those forming biofilms, can be one of the possible reasons for such impairment [196]. Numerous studies report the prevalence of biofilm in chronic cutaneous wounds at anywhere between 20 and 100% [197]. In the oral cavity, several chronic conditions associated with bacterial dysbiosis and resulting in poor healing, namely periodontitis [198] and mucosal lichen planus [199], have been found to increase the risk of developing oral SCC. In oral SCC, two prominent oral pathogens, *Porphyromonas gingivalis* and *Fusobacterium nucleatum,* can promote tumour progression in mice [200]. It has been observed that these infections lead to the proliferation of oral cancer stem cells [201] and also significantly contribute to the increased invasivity of SCC via promotion of EMT [202]. There is also evidence that the microbiome can adversely interact with cancer therapies [203].

Surprisingly, bacterial colonisation is not always associated with activation of the immune response. In triple-negative breast cancer, it has been shown that *Fusobacterium nucleatum* not only colonised these tumours but was also found to be responsible for the immunosuppressive tumour microenvironment and tumour metastasis [204]. A similar observation was confirmed in, e.g., colorectal cancer [205]. Once the infection was cleared, e.g., with metronidazole, dead intratumoral bacteria were transformed into immunopotentiators in immunotherapy via the release of pathogen-associated molecular patterns, influencing the subsequent maturation of dendritic cells and subsequent T cell infiltration. Evidence of the multifaceted intratumoural microbiome and its roles is growing exponentially [206,207,208,209].

However, these results should be interpreted cautiously. Several studies pointed out that depletion of gut microbiota with an antibiotic treatment can compromise the anticancer effect of anti-CTLA-4 therapy in mouse models of sarcoma, melanoma, and colon cancer [210]. Similarly, it has been demonstrated that antibiotic administration also compromises the clinical benefits of PD-1-based immunotherapy, both in mice and in humans [211]. Restoration of intestinal microbiota as an adjuvant factor in cancer therapy was investigated in clinical studies [212,213], with somewhat positive outcomes reported. However, it is too early to draw definitive conclusions from these preliminary data acquired on small published cohorts.

## 5. Systemic Effects of Tumours

The terminal stage of malignant disease is characterised by wasting, cachexia, and psychological changes, including loss of appetite. These life-threatening events seem to be triggered by mediators of cellular communication within the tumour system, particularly a high level of IL-6, which activates STAT3 even in normal cells. This process leads to altered metabolism in white adipose tissue, skeletal muscle, and hepatocytes [214].

Focusing on the brain, an animal experiment showed that IL-6 activates neurons in the *area postrema* of the brain and, through signalling to other brain regions, causes dopamine deficiency in the *nucleus accumbens*, leading the animal to become apathetic and refuse food [215]. Similar conditions, such as apathy, depression, and loss of appetite, are also observed in patients with malignant diseases [216,217,218]. In patients with malignant tumours experiencing these symptoms, we assume the same mechanism described above is at work. Patients with malignant tumours often also suffer from a diverse range of paraneoplastic syndromes, which encompass a variety of complications from cutaneous to neurological [192]. These conditions are also triggered by bioactive factors produced by tumours.

In humans, mood disorders are associated with chronic low-grade systemic (sterile) inflammation, with increased plasma levels of pro-inflammatory cytokines like TNF-α and IL-6, which regulate mood behaviour and cognition by influencing neurotransmitter levels [219]. However, the mechanisms underlying this enhanced inflammation are not well understood. In intestinal tumours, the dysbiosis of microbiota can significantly interfere with the immune response and digestion, but also with cortisol regulation and control of neurotransmitters and their metabolism, including serotonin, dopamine, noradrenaline, and gamma-Aminobutyric Acid (GABA) [220]. These neuromediators can not only act locally, but they can also enter circulation. There is a growing body of evidence indicating that modulation of gut microbiota exerts a modulatory influence on depression and exhibits therapeutic promise [221]. Relevant for head and neck cancers, depressive symptoms evaluated in these patients were significantly associated with inflammation. Moreover, both depression and inflammation were associated with early mortality [222].

In many malignant diseases, a broad spectrum of alterations in body metabolism has been documented [223]. In this regard, the liver appears to be a crucial target that warrants closer attention. In recent years, tumour-derived extracellular vesicles were identified as potent mediators of intercellular communication within the cancer microenvironment and also over long distances across the whole organism [224,225]. More closely, exosomes are also crucial in cancer-induced hepatic reprogramming. Of note, this dysregulation of hepatic functions happens commonly even in tumour-free livers of patients who developed extrahepatic metastasis [226]. Liver accumulation of cholesterol, triglycerides, and ceramides is linked to non-alcoholic fatty liver disease [227]. It seems that extracellular vesicles primarily act on Kupffer cells [228] via an IL-1-dependent mechanism. Notably, tumour-originated extracellular vesicles worsen the adverse effects of chemotherapy, including bone marrow suppression and cardiotoxicity. This can be linked to poor tolerance of chemotherapy and a worsened prognosis [226].

Frequently neglected, adipose tissue is a remarkably metabolically active endocrine organ of the human body. It is also a highly relevant topic in cancer. It is well documented that obesity, the abnormal or excessive expansion of white adipose tissue, is a common root for several malignancies [229,230]. Among the adipokines released from this tissue, leptin is recognised as one of the most important mediators of obesity-associated cancers [231]. There is well recognised pro-inflammatory signalling between cancer cells and adipocytes via the insulin/IGF-1 axis and adipokines [232].

Conversely, it is well recognised that exercise can reduce body weight and also reduce obesity-associated cancer risk. Mechanistically, the underlying mechanisms behind the positive effects of exercise remain largely poorly understood, but it was suggested that fibronectin type III domain-containing protein 5 (FNDC5)/Irisin, a hormone released from exercising muscle, could be responsible for this [233]. Further, upon exercise, skeletal muscles react by secreting various types of myokines, namely IL-6, interleukin-8, interleukin-15, brain-derived neurotrophic factor (BDNF), and irisin. These mediators can improve obesity-induced inflammation by stimulating lipolysis of adipose tissues, promoting glucose uptake [234]. This also notably accelerates the browning of white fat. Brown fat tissue is metabolically very different from white adipose tissue in structure and also in function [235].

In a mouse model, it was observed that exposure of tumour-bearing mice to cold conditions markedly inhibited the growth of various types of solid tumours [236]. Mechanistically, it was suggested that upon this cold exposure, brown adipose tissue was activated, which substantially decreased blood glucose and impeded glycolysis-based metabolism in cancer cells. Conversely, feeding on a high-glucose diet under cold exposure restored tumour growth. Notably, in a small pilot human study, mild cold exposure activated brown adipose tissue and mitigated glucose uptake in the tumour tissue [236]. As glucose uptake is essential for cancer glycolysis, this opens the path to novel therapeutic interventions [237].

However, there is another aspect of adipose tissue—the end-stage, cachexia [238]. Cancer cachexia is quite commonly seen in advanced cancer patients. In these individuals, weight loss is a characteristic symptom of cancer cachexia, along with decreased skeletal muscle mass. Despite extensive research, the pathogenesis of cachexia has not been fully understood until now. The onset of cachexia and its gradual further progression are clinically evident. As mentioned earlier, cancer-associated systemic inflammation is one of the critical pathophysiologies of cancer cachexia, resulting in weight loss due to degradation of skeletal muscle and adipose tissue and suppression of appetite. Prevention of weight loss by early nutritional intervention and exercise may also lead to, at least in some patients, continuation of cancer treatment and a more favourable prognosis. In mouse models, inflammatory cytokines such as IL-6 were elevated in mice with cachexia. In mice in which these inflammatory cytokines are suppressed, weight loss did not occur even when cancer progressed [239]. The importance of this aspect in oncology and its relevance to clinical interventions was acknowledged in the Consensus on the definition and diagnosis of sarcopenia (EWGSOP2) and the Global Leadership Initiative on Malnutrition (GLIM) criteria [240,241]. This is of special concern in the elderly, in whom sarcopenia, malnutrition, and cancer cachexia are highly prevalent due to ageing and can adversely impact prognosis in cancer treatment [242]. It is obvious that complex and multifaceted metabolic crosstalk mechanisms represent challenges, but also opportunities, in the treatment of these devastating neoplastic diseases [243].

## 6. Conclusions

HNSCCs represent a highly heterogeneous group of tumours, both oncologically and biologically, with different etiopathogenesis, often partly based on the presence of HPV infection. HPV-positive tumours tend to have a better prognosis, and their pathogenesis involves the viral oncoproteins E6 and E7. Conversely, HPV-negative tumours usually have a worse prognosis and are often linked to mutations in tumour suppressor genes and reduced p16 expression. These differences significantly influence prognosis and the therapeutic response.

A tumour (including HNSCC) is a complex ecosystem comprising tumour and nontumour cells, with mutual regulation distinct from the body’s normal control systems. Besides primary genetic alterations, the TME plays a vital role in tumour development. The TME includes not only tumour cells but also CSCs, CAFs, immune cells, blood vessels, ECM, and signalling molecules.

CSCs are a key subpopulation with a high capacity for tissue regeneration—including tumour tissue—along with invasiveness and resistance to therapy. They are likely a major cause of recurrence and metastasis. Understanding tumour evolution requires recognising its Darwinian trajectory, driven by selection pressures within the TME.

CAFs play a crucial role in the TME. They are highly heterogeneous and originate from various sources. CAFs can influence the behaviour of surrounding tumour cells and normal cells. They also produce the ECM (e.g., tenascin-C), remodel their environment, and affect angiogenesis and immune responses. Their paracrine effects contribute to tumour progression, EMT, and changes in cell phenotype.

The EMT (epithelial–mesenchymal transition) and its reverse process, MET (mesenchymal–epithelial transition), are vital in the migration and colonisation of distant tissues by tumour cells, which is an important aspect of metastasis—a key process in malignant tumours. Some tumour cells may employ alternative migration mechanisms, such as amoeboid movement. It is important to note that CSCs can survive without attachment to the matrix (they are anoikis-resistant). This facilitates the survival of CSCs in circulation and subsequent metastasis formation.

Intercellular signalling within the TME is essential for the specific functions of cells and mechanisms of tumourigenesis. Molecules like IL-6, IL-8, CXCL1, MIF, or TGF-β are involved in inflammatory, immunoregulatory, and anticancer mechanisms. Cytokines such as IL-6 play a key role in the systemic effects of cancer, including cachexia and neuropsychiatric symptoms. Inhibition of these molecules in preclinical studies shows therapeutic potential; however, transitioning to clinical practice has yet to prove effective.

During tumourigenesis and metastasis, immune cells, especially TAMs, support tumour progression. Targeted oncological therapies inhibiting immune checkpoints (e.g., PD-1/PD-L1) appear promising. However, they are only effective in patients with so-called “hot” tumours. Conversely, they tend to be ineffective in cases of “cold” tumours.

Blood supply through neovascularisation is essential for tumour growth. It is common for hypoxia zones to form within the tumour. Hypoxia stimulates the expression of specific factors, such as HIF and VEGF, which promote angiogenesis and tumour aggressiveness. Furthermore, a hypoxic environment contributes to a poorer response to therapy.

Finally, it is important to note that tumours and wounds share several fundamental similarities—both involve fibroblast activation, ongoing inflammation, granulation tissue formation, and secretion of growth factors. A tumour can be viewed as a state of chronic wound healing that has escaped physiological regulation.

Overall, effective oncological therapy should target not only tumour cells but also the dynamic TME, including CAFs, CSCs, immune cells, and intercellular signals. However, achieving this requires a thorough understanding of the patterns of TME function.

## Data Availability

No new data were created.

## Supplementary Materials

The following supporting information can be downloaded at https://www.mdpi.com/article/10.3390/ijms26188844/s1.

## Abbreviations

The following abbreviations are used in this manuscript:

a-SMA	a-Smooth Muscle Actin
ACT	Adoptive Cytotoxic T cell transfer
apCAFs	Antigen-Presenting Cancer-Associated Fibroblasts
BMP-4	Bone Morphogenetic Protein 4
CAFs	Cancer-Associated Fibroblasts
CCL	Chemokine (C-C motif) Ligand
CD	Cluster of Differentiation
CDK4-6	Cyclin-Dependent Kinase 4-6
CDKN2A	Cyclin-Dependent Kinase inhibitor 2A
CSCs	Cancer Stem Cells
CTLA-4	Cytotoxic T lymphocyte-associated protein 4
CXCL	C-X-C motive chemokine ligand
CXCR	C-X-C chemokine receptor
DPPIV	Dipeptidyl-peptidase IV
ECM	Extracellular Matrix
EGF	Epidermal Growth Factor
EMT	Epithelial–Mesenchymal Transition
FAP	Fibroblast Activation Protein
FGF	Fibroblast Growth Factor
FUS	Fused in Sarcoma protein
FUSIP1	FUS-Interacting Protein 1
Gal-1	Galectin-1
gp130	Glycoprotein 130
HGF	Hepatocyte Growth Factor
HIF	Hypoxia-Inducible Factor
HNSCC	Head and Neck Squamous Cell Carcinoma
HPV	Human Papillomavirus
iCAFs	Inflammation Cancer-Associated Fibroblasts
ICIs	Immune-Checkpoint Inhibitors
IGF-2	Insulin-like Growth Factor-2
IL-	Interleukin-
JAKs	Janus Activated Kinases
MAP3K2	Mitogen-Activated Protein Kinase Kinase Kinase 2
matCAFs	Matrix Cancer-Associated Fibroblasts
M-CSF	Macrophage Colony-Stimulating Factor
MDSCs	Myeloid-Derived Suppressor Cells
MET	Mesenchymal–Epithelial Transition
MIF	Macrophage Migration Inhibitory Factor
myCAFs	Myofibroblastic Cancer-Associated Fibroblasts
NF- κB	Nuclear Factor kappa-light-chain-enhancer of activated B cells
NG2	Neuron-Glial antigen 2
PD1-L	Programmed cell death protein 1 ligand
PD1-R	Programmed cell death protein 1 receptor
PDGFR	Platelet-Derived Growth Factor receptor
pRb	Retinoblastoma protein
PTPLAD1	Protein Tyrosine Phosphatase-Like A Domain Containing 1
RICs	Regeneration-initiating cells
ROCs	Regeneration-organising cells
SLC25A40	Solute Carrier Family 25 Member 40
SPIN1	Spindlin 1
STAT3	Signal Transducer and Activator of Transcription 3
TAMs	Tumour-Associated Macrophages
TGFβ	Transforming Growth Factor β
TME	Tumour Microenvironment
TNFa	Tumour Necrosis Factor α
TNFb	Tumour Necrosis Factor β
Treg	Regulatory T cells
TRIM23	Tripartite Motive Containing 23
VEGF	Vascular Endothelial Growth Factor
