# Supplementary figures and images for: Cold, Hot, and Lethal—The Tumour Microenvironment and the Immunology of Head and Neck Squamous Cell Carcinoma
Source: Int J Mol Sci. 2025 Sep 11;26(18):8844. doi: 10.3390/ijms26188844 (PMC12469655; doi:10.3390/ijms26188844)

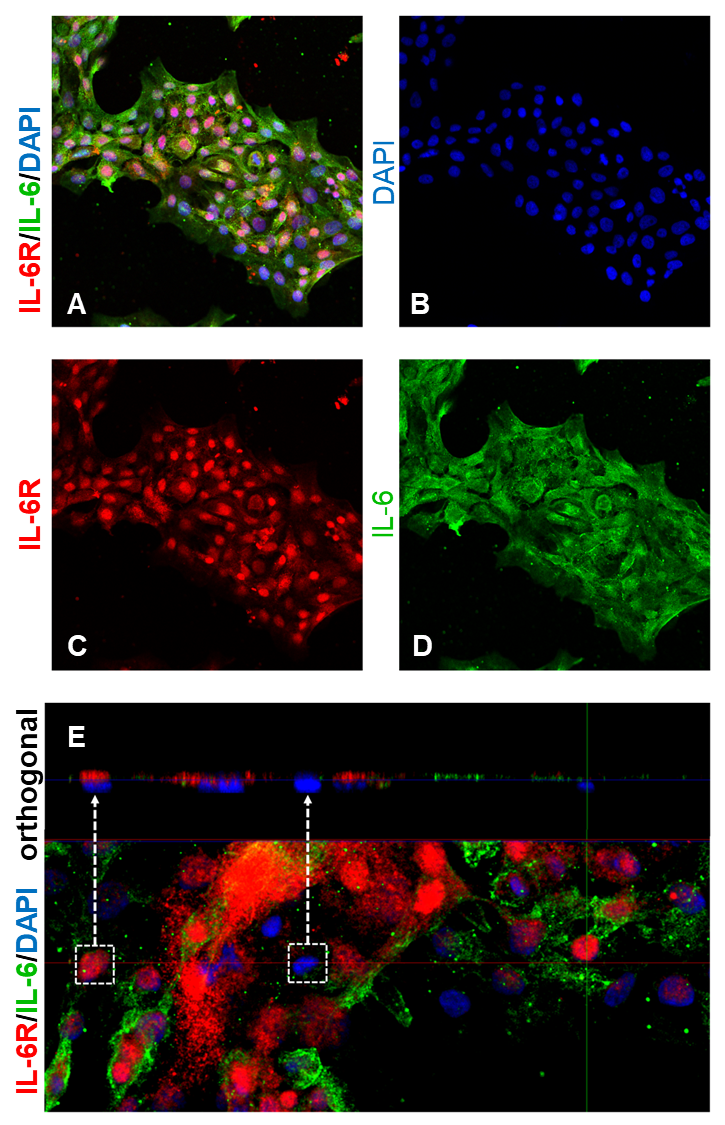

Supplement: Supplementary file 1 [file ijms-26-08844-s001.zip › Figure S1.tif]
